# An Assessment of the Performance Limitations of the Integrated Quantifiler^TM^ Trio-HRM Assay: A Forensic Tool Designed to Identify Mixtures at the Quantification Stage

**DOI:** 10.3390/genes15060768

**Published:** 2024-06-12

**Authors:** Chastyn Smith, Sarah J. Seashols-Williams, Edward L. Boone, Tracey Dawson Green

**Affiliations:** 1 Integrative Life Sciences, Virginia Commonwealth University, 1000 Cary Street, Richmond, VA 23284, USA; 2 Department of Forensic Science, Virginia Commonwealth University, 1015 Floyd Avenue, Richmond, VA 23284, USA; sseashols@vcu.edu (S.J.S.-W.); tcdawson@vcu.edu (T.D.G.); 3 Department of Statistical Sciences & Operations Research, Virginia Commonwealth University, 1015 Floyd Avenue, Richmond, VA 23284, USA; elboone@vcu.edu

**Keywords:** qPCR, forensic samples, HRM, mixture screening, prediction modeling, developmental validation

## Abstract

Although guidelines exist for identifying mixtures, these measures often occur at the end-point of analysis and are protracted. To facilitate early mixture detection, we integrated a high-resolution melt (HRM) mixture screening assay into the qPCR step of the forensic workflow, producing the integrated Quantifiler^TM^ Trio-HRM assay. The assay, when coupled with a prediction tool, allowed for 75.0% accurate identification of the contributor status of a sample (single source vs. mixture). To elucidate the limitations of the developed qPCR-HRM assay, developmental validation studies were conducted assessing the reproducibility and samples with varying DNA ratios, contributors, and quality. From this work, it was determined that the integrated Quantifiler^TM^ Trio-HRM assay is capable of accurately identifying mixtures with up to five contributors and mixtures at ratios up to 1:100. Further, the optimal performance concentration range was found to be between 0.025 and 0.5 ng/µL. With these results, evidentiary-like DNA samples were then analyzed, resulting in 100.0% of the mixture samples being accurately identified; furthermore, every time a sample was predicted as a single source, it was true, giving confidence to any single-source calls. Overall, the integrated Quantifiler^TM^ Trio-HRM assay has exhibited an enhanced ability to discern mixture samples from single-source samples at the qPCR stage under commonly observed conditions regardless of the contributor’s sex.

## 1. Introduction

Currently, in the conventional forensic DNA workflow, the number of contributors in an evidentiary DNA sample is unknown until the end-point of analysis. Due to this, mixtures are not revealed until STR profile generation, at which point, mixtures can still be difficult to interpret due to allele sharing and allele masking [1,2]. These challenges can lead to delays in casework due to the time spent on profile explication and occasional reanalysis. The DNA Advisory Board (DAB) issued quality assurance (QA) standards in 2000 for U.S. forensic DNA labs that included recommendations for mixture interpretation; however, these recommendations were made with simple two-person mixtures in mind, derived mostly from the perspective of sexual assault cases [3]. Yet, within the past several years, the submission of evidentiary samples with more complex mixtures (samples that contain biological material from >2 contributors) has increased [4]. Although these guidelines have been updated and additional guidelines for the interpretation of these complex samples have been issued over the past 24 years, other factors like low-template DNA and allele sharing have increased the intricacy of complex mixtures [5,6,7,8].

Similarly, due to the advancement of forensic STR kits and DNA analysis instrumentation, namely capillary electrophoresis (CE), the submission of “touch” or “contact” samples has also increased [9,10]. As they are often derived from surfaces or items that could be handled or touched by numerous individuals, they often contain traces of genetic material from multiple contributors. Thus, this results in complex mixtures that may contain minute amounts of DNA from one or all contributors or can result in complex mixtures where the contributions of each individual vary, producing different mixture ratios. In a study conducted by Hanned et al., out of 8470 casework profiles examined, 39% were derived from touch samples. Of the touch samples examined, 51% contained DNA from more than two contributors [11]. Although STR-CE typing is the gold standard for human identification, this method falls short in the detection and deconvolution of complex mixtures, primarily those that contain major to minor contributor ratios greater than 10:1, and low-template/-quality DNA [12,13,14]. Increased submissions of these types of evidentiary samples (containing low-quantity and -quality DNA) combined with a workflow that relies on the end-point identification of mixtures leads directly to backlogs and lower case success rates [1]. Thus, a screening assay that could provide information about the nature of a sample’s contributors, particularly earlier in the workflow, could help better determine how to proceed with processing a sample *prior* to STR amplification. Having access to this information would allow analysts to enact enhanced detection methods for these complex samples that would increase the likelihood of generating a successful profile on the first attempt, enabling a more straightforward mixture deconvolution and shorter turnaround times.

To address this need, researchers have previously reported the development of two high-resolution melt (HRM) mixture screening assays that have been integrated into the qPCR step of the forensic workflow using multiple real-time instruments [15,16,17]. The developed assays incorporate two co-amplified short tandem repeats (STRs: D5S818 and D18S51), along with a DNA-binding dye, into two different commercially available human DNA quantification kits: Investigator Quantiplex^®^ (QIAGEN, Hilden, Germany) and Quantifiler^TM^ Trio (Thermo Fisher Scientific, Waltham, MA). Amplification is followed by a high-resolution melt (HRM) cycle and data analysis [16,17,18]. When coupled with sequential statistical analysis by three machine learning prediction algorithms [support vector machine (SVM) linear, SVM radial, and linear discriminant analysis (LDA)], the assay is capable of accurately predicting the contributor status (single source vs. mixture) of unknown samples [17,18,19]. The initial testing of these assays included over 120 unique single-source samples and two-person 1:1 mixtures comprising seven different genotypes of interest (for each STR locus included). A portion of the samples, the “training” standards, were used to train the machine learning models, while 56 single-source samples and up to 16, 1:1, two-person mixtures were treated as an unknown “validation” or test samples [16,17,18,19].

Initially, the integrated Quantiplex^®^-HRM assay was developed and optimized for use on the Rotor-Gene^®^ Q for proof-of-concept testing. This assay was able to accurately distinguish between single-source and mixture samples 89.4% of the time using the best performing prediction models—SVM radial and SVM linear for D5S818 and D18S51, respectively [17]. The assay was then assessed on a newer qPCR platform, QuantStudio^TM^ (Thermo Fisher Scientific), which is supported for use with several common forensic DNA quantification kits [20,21]. On the QuantStudio^TM^ 6 Flex, 87.9% of the single-source and mixture samples were accurately distinguished using the best performing prediction models—SVM linear and SVM radial for D5S818 and D18S51, respectively [18]. However, the Investigator Quantiplex^®^ qPCR kit is not commonly used in operational forensic DNA laboratories as it employs the use of only one human DNA (hDNA) target, which can limit sample information [22]. Thus, the HRM components were then modified to function within the Quantifiler^TM^ Trio qPCR kit, which is more informative and more widely used in the forensic community [23]. Despite the modifications made to the assay, the integrated Quantifiler^TM^ Trio-HRM assay produced hDNA quantification values, M:F ratios, degradation indices, and DNA profiles concordant to those obtained when the standard Quantifiler^TM^ Trio reaction was employed [16]. Further, the integrated Quantifiler^TM^ Trio-HRM assay was able to correctly classify 79.2% of the single-source and mixture samples [16]. However, for this assay, the best performing prediction models for D5S818 and D18S51 were SVM radial and SVM linear, respectively [16]. Unfortunately, further evaluation revealed markedly reduced accuracies when the training standards and test samples were amplified using different lots of the Quantifiler^TM^ Trio kit. Thus, an expanded training set that included replicates tested across multiple lots was developed and used going forward. When used to train the prediction models, the expanded consensus training set produced accuracies similar to those previously reported (75.0% overall). Further, previously reported data [24] indicated that this assay coupled with the machine learning models described could effectively identify samples even when their genotypes were not seen in the dataset used to train the models. Taken together, these data demonstrated the robust capability of this approach for the successful prescreening identification of mixtures, performing at rates well beyond the probabilities expected by random chance classification (6.25%).

For a finalized assay that has been proven to be capable of distinguishing mixture samples from single-source samples at the qPCR stage, the limitations of the assay would need to be defined; this step is required of all new forensic methods prior to implementation and these experiments are typically included as a part of a developmental validation [5,25]. Thus, a select group of essential developmental validation studies were conducted in order to assess a variety of sample conditions and to determine the performance limitations of the newly developed integrated Quantifiler^TM^ Trio-HRM assay. Importantly, this testing must include an exploration of the reproducibility and sensitivity of the assay. Further, given that all the previous mixture samples tested for development and proof-of-concept were relatively simple—high-quality DNA from two persons mixed together at even ratios—additional testing using altered conditions would be needed. This includes testing mixtures with a varying number of contributors combined at varying ratios across a broad spectrum and testing low-quality, forensically challenged, and relevant samples [7]. With these validation studies completed and the development of a user-friendly web interface, the application would be primed to deploy to partner forensic practitioner laboratories for external testing. If successful, this tool has the potential to provide forensic examiners with a powerful method for the screening and triage of evidence items prior to the workflow end-point.

## 2. Materials and Methods

### 2.1. Sample Collection and STR Profile Generation

Previously collected buccal swab samples were obtained from the VCU forensic biological sample registry and were in accordance with the approved VCU Institutional Review Board, Human Subjects Research Protocol (VCU-HM20002931). Sample DNA was purified using a Qiagen QIAcube liquid extraction robot with the standard manufacturer’s “Buccal Swab Spin QIAcube Protocol” and QIAamp^®^ DNA Blood Mini kit reagents (Qiagen, Hilden, Germany). Sample DNA extracts were quantified using half-reactions of the Investigator Quantiplex^®^ kit on the Rotor-Gene^®^ Q (QIAGEN) following the manufacturer’s recommended protocols. Sample STR reference profiles were developed by amplifying 1 ng of DNA extract from each sample with the AmpFLSTR^®^ Identifiler^®^ PCR amplification kit (Thermo Fisher Scientific, Waltham, MA, USA) on a GeneAmp 9600 thermal cycler (PerkinElmer, Waltham, MA, USA). The 15 µL reaction consisted of 5.7 µL of PCR Reaction mix, 2 µL of Primer set, 2.1 µL Tris-EDTA (TE), 0.2 µL of AmpliTaq^TM^ Gold Polymerase (5 U/µL) (Thermo Fisher Scientific), and 5 µL of template DNA. Thermal cycling parameters were as follows: activation at 95 °C for 11 m followed by 28 cycles of 94 °C denaturation for 60 s, 59 °C annealing for 60 s, and 72 °C elongation for 60 s, finished with a 60 °C final extension for 90 m. Amplified STR products were separated and detected on an Applied Biosystems^TM^ 3130 Genetic Analyzer (Thermo Fisher Scientific) using a 36 cm capillary array and a 10 s injection with an analytical threshold of 75 relative fluorescent units (RFUs). For capillary electrophoresis analysis, 1.5 µL of amplified DNA or 1 µL of allelic ladder was mixed with 0.1 µL of GeneScan^TM^ 500-LIZ^TM^ size standard (Thermo Fisher Scientific) and 12 µL of Hi-Di^TM^ formamide (Thermo Fisher Scientific). STR profiles were analyzed using GeneMapper^TM^ ID software v4.1 (Thermo Fisher Scientific). Samples that expressed genotypes of interest at the D5S818 (D5) [(10,11), (11,11), (11,12), (11,13), (12,12), (12,13) and (13,13)] and D18S51 (D18) [(12,13), (12,14), (12,15), (12,16), (13,14), (13,16), and (14,15)] loci were used for all experimental studies (*n* = 124).

These DNA extracts were used for all studies detailed below. For single-source samples, 2.0 µL from each DNA extract was used. All of the following proportional mixtures were made by combining 0.2 ng/µL or 0.4 ng/µL of the DNA extract from each contributor using the quantification values obtained as described above.

### 2.2. Integrated Quantifiler^TM^ Trio-HRM Assay

Each experimental sample used was evaluated with the integrated Quantifiler^TM^ Trio-HRM assay using the QuantStudio^TM^ 6 Flex Real-Time PCR System software v1.3 (Thermo Fisher Scientific) following the finalized formal protocol [16]. The 16 μL volume reaction included 5.8 µL of Quantifiler^TM^ Trio Primer mix, 7.2 µL of Quantifiler^TM^ Trio THP Reaction mix, 0.10 µL of 240.45µM and 100µM D5 and D18 primers, respectively, 0.63 µL of 128µM SYTO^TM^ 64, and 2.0 µL of sample DNA or standard sample DNA input (as recommended by the manufacturer). Data analysis settings included baseline cycle start and end values of 3 and 17, respectively, for all targets and a threshold of 0.4, 0.08, and 0.1 for IPC, large autosomal, and small autosomal/Y targets, respectively. Sample amplification followed the manufacturer’s recommended protocol with an added transition cycle [72 °C for 2 m, 95 °C for 20 s, 55 °C for 20 s, and 56 °C for 2 m (with a 1.6 °C/s ramp rate)] and then a high-resolution melt from 60 °C to 95 °C. Data were collected using the continuous setting set at 0.015 °C/s. For each tested sample, the negative derivative melt data were exported from the QuantStudio^TM^ 6 Flex Real-Time PCR System software v1.3, organized by STR locus, converted to a CSV file, and then imported into RStudio^®^/R statistical software v4.3.1 (^©^The R Foundation, Vienna, Austria) for analysis using three different machine learning prediction modeling tools: LDA, SVM linear, and SVM radial. Each prediction model was trained using the expanded consensus training set that consisted of 114 unique single-source DNA samples with either D5S818 or D18S51 genotypes of interest along with 16 1:1 two-person mixtures. For each new Quantifiler^TM^ Trio kit lot received, the training samples were re-tested with the integrated Quantifiler^TM^ Trio-HRM assay. The HRM data from each run, along with data from 10 additional mixtures (containing different genotypes, not previously included), were assembled into one reference dataset. In its entirety, the consensus training set contained over 300 single-source D5S818 and D18S51 melt curve profiles and 58 1:1 two-person mixture melt curve profiles tested using up to four different lots of Quantifiler^TM^ Trio kit components.

Previous assessments of HRM data from the integrated Quantifiler^TM^ Trio-HRM assay using the expanded consensus training dataset recommended the SVM radial model (imbedded in R software) as the best performing statistical prediction tool. Consequently, the SVM radial model was used to predict the contributor nature (single source vs. mixture) of each experimental sample tested. Classification categories used included the 7 known genotypes included for D5S818 and the 7 known genotypes included for D18S51 as well as a mixture category for each locus tested; samples that were classified as a specific genotype at D5S818 or D18S51 were indicated as single-source at that locus while those that were classified as a mixture were indicated as a mixture (Table 1). If either STR locus was classified as a mixture for a given sample, then the final classification for that sample was indicated as a mixture; those that were predicted as single-source at both loci tested were indicated as single-source. Combined single-source prediction accuracy was determined by calculating the total number of samples classified as a single source (regardless of the predicted genotype) divided by the total number of known single-source samples tested in that set. Similarly, the combined mixture prediction accuracy was determined by dividing the number of mixture samples correctly classified by the total number of known mixtures tested in that data set. Finally, the number of samples that were classified accurately was divided by the total number of samples tested in order to determine the overall prediction accuracy.

### 2.3. Validation Testing

#### 2.3.1. Reproducibility

To assess the reproducibility of the integrated Quantifiler^TM^ Trio-HRM assay, 17 unknown samples (7 single-source and 10 two-person 1:1 mixture samples) that had been previously assessed in the integrated Quantifiler^TM^ Trio-HRM assay were re-tested as described above; however, data were analyzed in R software using *three* machine learning models—SVM radial, SVM linear, and LDA. To limit analyst- and experience-associated variation, the same analyst was used to set up all reactions for this study and this analyst had extensive experience with this assay (~4 years). Single-source vs. mixture prediction accuracies were calculated (as described above) using the output from each model. To assess the inter-run reproducibility, the prediction data obtained from each sample using the SVM radial model (previously recommended) were compared to the data obtained from a previous run. The reproducibility was determined by dividing the number of samples that resulted in identical predictions (single source or mixture) by the total number of samples tested. Similarly, the intra-run reproducibility was assessed by testing 18 unknown samples (8 single-source and 10 two-person 1:1 mixtures) in duplicate on a single run followed by SVM radial analysis and comparison of each dataset separately (as described above). After comparison of the SVM radial model data, data from the SVM linear and LDA models were closely examined and the best performing models at each STR locus tested were identified and those numbers were reported.

#### 2.3.2. Sensitivity

To determine the lower limit of detection of the integrated Quantifiler^TM^ Trio-HRM assay, five single-source samples and five two-person 1:1 mixture samples were diluted and/or concentrated to the following final concentrations (ng/µL): 60, 50, 5, 0.5, 0.05, 0.025, and 0.01. A total of 70 samples were tested using the final integrated Quantifiler^TM^ Trio-HRM assay, as described above, and data were analyzed with three machine learning models (SVM radial, SVM linear, and LDA) in R software. Prediction accuracies were calculated, as described above, and accuracies from the best performing model at each locus were reported. Single-source, mixture, and overall prediction accuracies were determined, as described above.

#### 2.3.3. Increased Number of Contributors

In order to assess the ability of the integrated Quantifiler^TM^ Trio-HRM assay to identify more complex mixtures, 5 of the 16 previously tested 1:1 two-person mixtures were remade to include a 3rd, 4th, and 5th contributor; this generated a total of 31 mixture samples for testing. For each mixture, all contributors were included at equal ratios. Further, contributors within a single mixture sample included those with different genotypes at either the D5S818 or D18S51 locus. Samples were tested using the final integrated Quantifiler^TM^ Trio-HRM assay, as described above, and data were analyzed with three machine learning models (SVM radial, SVM linear, and LDA) in R software. Prediction accuracies were calculated, as described above, and accuracies from the best performing model at each locus was reported for each experimental group (two-, three-, four-, and five-person mixtures). Additionally, overall prediction accuracy across all experimental groups was determined, as described above.

#### 2.3.4. Mixture Ratios

In order to assess the ability of the integrated Quantifiler^TM^ Trio-HRM assay to identify more complex mixtures, 10 of the previous 16 1:1 two-person mixtures were remade at various ratios (1:2, 1:5, 1:10, 1:20, 1:50, 1:100, 100:1, 50:1, 20:1, 10:1, 5:1, and 2:1). For the 1:20/20:1, 1:50/50:1, and 1:100/100:1 groups, we were unable to remake two mixtures due to the exhaustion of a sample. Samples were tested using the final integrated Quantifiler^TM^ Trio-HRM assay, as described above, and data were analyzed with three machine learning models (SVM radial, SVM linear, and LDA) in R software. Prediction accuracies were calculated, as described above, and accuracies from the best performing model at each locus were reported for each experimental ratio group. Additionally, overall prediction accuracy across all experimental groups was determined, as described above.

#### 2.3.5. Mock Forensic/Evidentiary Samples

In order to test the integrated Quantifiler^TM^ Trio-HRM assay’s ability to accurately predict the contributor status of forensic-like samples, mock evidence samples were tested. DNA extracts from a previous study were utilized; this included six venous blood swabs taken from cotton swatches, four–five touch swabs taken from worn t-shirts, and nine contact swabs taken from used keyboards for a total of 20 mock samples; all samples were collected using Bode BioSafe^TM^ swabs [26]. All samples used in this study were aged at different temperatures for different time periods in order to accelerate the aging process [26]. Each temperature and time period combination was converted into simulated days (SDs), which represented the theoretical number of days the samples would have been stored at 25 °C. DNA profiles were developed via STR-CE analysis to determine the true contributor status of each sample. Samples determined to be mixtures contained 3–6 alleles at two or more loci.

Samples were tested using the final integrated Quantifiler^TM^ Trio-HRM assay, as described above, and data were analyzed with three machine learning models (SVM radial, SVM linear, and LDA) in R software. The degradation index (DI) values and DNA quantification values obtained from the integrated Quantifiler^TM^ Trio-HRM assay were compared to those values obtained from testing using the standard Quantifiler^TM^ Trio assay for 17 of the samples tested. For standard quantification, the manufacturer’s recommended protocols were utilized along with the Quantifiler^TM^ Trio kit, but samples were tested using half-volume reactions. DI values for each sample were calculated by dividing the small autosomal quantification values by the large autosomal quantification values; a mean was then calculated for each experimental group. Prediction accuracies were calculated, as described above, and accuracies from the best performing model at each locus was reported for each experimental ratio group. Additionally, overall prediction accuracy across all mock samples was determined, as described above.

## 3. Results and Discussion

### 3.1. Reproducibility

As reported, among the machine learning tools tested, SVM radial trained with an expanded consensus dataset produced the most promising prediction results when the samples were characterized using the integrated Quantifiler^TM^ Trio-HRM assay [24]. Accordingly, the reproducibility of the assay was initially evaluated using only the SVM radial model. For the inter-run reproducibility, 71.4% of the single-source samples produced reproducible predictions, whereas 30.0% of the mixtures tested produced identical predictions across runs (Table 2). When the reproducibility was assessed for replicates tested on the same run (intra-run), 57.1% of the single-source samples and 70.0% of the mixture samples produced identical predictions. Overall, when utilizing the previously recommended model (SVM radial), the inter-run reproducibility of the integrated Quantifiler^TM^ Trio-HRM assay was found to be slightly lower than the intra-run reproducibility, as expected (47.1% vs. 64.7%, respectively).

When the other prediction algorithms were evaluated, however, differences in the performance were noted. In fact, the algorithm that best predicted the nature of the sample contributor varied with each new dataset tested. Consequently, the reproducibility was re-examined based upon the models that produced the highest prediction accuracies with each run. For the inter-run sample dataset, SVM linear and LDA were the best performing prediction accuracy models for D5S818 and D18S51, respectively. When the inter-run reproducibility was assessed using these models, the reproducibility was markedly increased, with 85.7% of the single-source samples and 40.0% of the mixture samples tested (58.8% overall) being predicted identically across both runs (Table 2). Although different models performed best for accuracy when the intra-run datasets were classified (LDA and SVM linear for D5S818 and D18S51, respectively), when those models were used to assess the intra-run reproducibility, the values were the same as those observed using only SVM radial. Given that the inter-run reproducibility of the integrated Quantifiler^TM^ Trio-HRM assay was improved when the best performing prediction accuracy models were examined, it is recommended that all three machine learning models investigated herein be used with the integrated Quantifiler^TM^ Trio-HRM assay for the classification of all unknowns going forward. For practical use, a consensus algorithm can be programed into the code that would take into consideration the prediction from all three prediction models and output a consensus prediction for the sample in question. This approach would provide the user with more consistent results and would reduce the potential for the mischaracterization of samples over time.

### 3.2. Sensitivity

**Figure 1:**
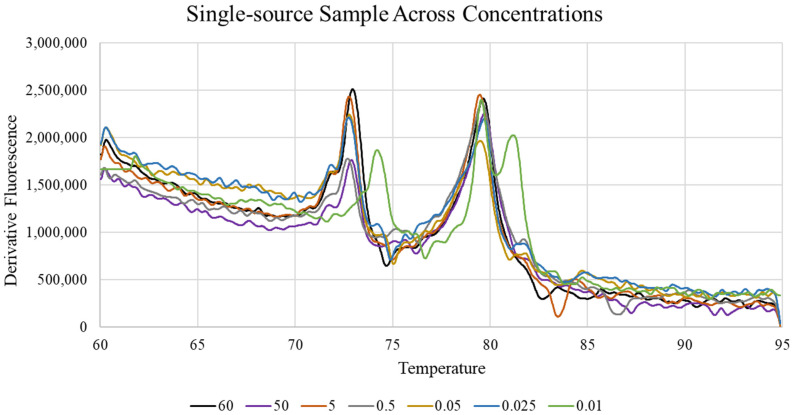
High-resolution melt curve of a single-source sample tested across a range of DNA concentrations (ng/µL).

The sensitivity of the integrated Quantifiler^TM^ Trio-HRM assay was assessed by testing single-source and mixture samples that ranged in DNA concentration from 0.01 to 60 ng/µL. For this study, 71.4% of all the tested single-source samples were accurately identified regardless of their concentration; however, all of the accurate classifications occurred with samples whose known DNA concentrations were above 0.025 ng/µL (Table 3). Further, substantial temperature range shifts were noted in the melt curve morphologies of the single-source samples whose DNA concentrations were lower than 0.025 ng/µL (Figure 1). Of the mixtures tested, 57.1% were accurately classified across all of the concentrations tested, with the most accurate sample predictions being among the samples with lower DNA concentrations (between 0.01 and 0.5 ng/µL) (Table 3). While mixture detection remains robust at very low DNA concentrations, when taken together, these data indicate that the integrated Quantifiler^TM^ Trio-HRM assay performs optimally for unknown samples with DNA concentrations between 0.025 ng/µL and 0.5 ng/µL. Unknown samples that are predicted to be mixtures with concentrations above this range and unknown samples that are predicted to be single-source samples with concentrations below this range should be interpreted with caution.

### 3.3. Increased Number of Contributors

The integrated Quantifiler^TM^ Trio-HRM assay was assessed for its ability to accurately identify complex mixtures, including those containing up to five contributors. Of over 30 samples tested, 87.1% were accurately predicted as either a single source or a mixture, regardless of the number of contributors included in the mixture (Table 4). As the number of contributors increased, the prediction accuracy increased, with 100.0% of the four-person and five-person mixtures being accurately classified as such (Table 4). This is to be expected, as the literature shows that HRM profiles of mixture samples are not a simple combination of the contributor’s alleles melting at the same time, but rather a recombination of alleles subsequently melting, thus producing melt profiles that are particularly distinct from single-source profiles of each contributor [27,28,29,30,31]. The data obtained in this study indicate that the integrated Quantifiler^TM^ Trio-HRM assay is robust in its ability to detect mixture samples, regardless of the number of contributors.

### 3.4. Mixture Ratios

As evidentiary samples include varying levels of each contributor’s DNA in a mixture sample, it was important to assess the ability of the integrated Quantifiler^TM^ Trio-HRM assay to accurately predict the nature of each sample’s contributor across a range of mixture ratios. Similar to what was previously observed, 75% of all the mixtures tested and 93.8% of the two-person 1:1 mixtures tested were accurately predicted as such [24]. In fact, mixture samples with contributor ratios down to 1:10 (and 10:1), including 1:2/2:1 and 1:5/5:1, were identified at high rates, consistent with those previously observed for 1:1 mixtures (Table 5). However, as the minor contributor dropped lower (at or below 1:20/20:1), the classification accuracies began to decrease (Table 5). This is not unexpected, given that the mixture sample’s melt curve would begin to more closely reflect that of the major contributor’s single-source morphology as the major contributor’s contribution to the mixture increases. However, it should be noted that the prediction accuracies were improved well over random chance even when the major contributor’s DNA was 100-fold higher than that of the minor contributor. Thus, the ability of this screening assay to predict low-level mixtures is better than that of the currently available end-point CE analysis. Additionally, although the standard Quantifiler^TM^ Trio assay can identify male/female mixtures at the quantification state, the integrated Quantifiler^TM^ Trio-HRM assay is the only assay that offers the ability to detect a mixture at this stage, regardless of the contributor’s gender.

### 3.5. Mock Forensic/Evidentiary Samples

With these performance limits elucidated, mock evidentiary blood and touch DNA samples were tested using the integrated Quantifiler^TM^ Trio-HRM assay. The total 20 samples utilized for testing were aged for 1–1440 simulated days at 25 °C, resulting in 10–100% of the expected STR alleles recovered upon traditional multiplex STR amplification and CE analysis [26]. The concentrations of these samples ranged from 0.0236 to 1.2087 ng/µL, with a mean small autosomal quantification value of 0.1741 ng/µL when tested with the standard Quantifiler^TM^ Trio assay or 0.2121 ng/µL when tested with the integrated Quantifiler^TM^ Trio-HRM assay (Table 6, *p* = 0.05). Of the 17 samples used for the quantification comparisons, 15 of the 17 samples had quantification values within the optimal range identified above. The average degradation indices were 1.64 or 1.17 when tested with the standard Quantifiler^TM^ Trio assay and the integrated Quantifiler^TM^ Trio-HRM assay, respectively (Table 6, *p* = 0.01). These data are in concordance with what was previously reported [16]; although there is a significant difference in the average DI values of each assay, this is not a practical difference as all DI values are less than 2 regardless of the assay, and would thus be treated identically in a forensic laboratory setting [32].

When assessing the prediction accuracies of the mock evidence samples, 12 out of the 19 samples tested were predicted correctly as either single-source or mixture samples, with 63.2% of the tested samples predicted accurately and 100% of all the mixture samples predicted accurately (Table 7). The results obtained for the mixture samples are consistent with the findings obtained from the sensitivity study for the samples in the optimal DNA concentration of 0.01–0.5 ng/µL. Further, as shown with the previously mentioned validation samples, the mock evidence mixtures were accurately classified regardless of the number of contributors in the DNA sample. Overall, this study demonstrated a remarkable ability of the assay to identify DNA samples that contain more than one contributor’s DNA (mixtures); further, while the single-source sample prediction accuracies are lower, every sample predicted as a single-source sample with this assay was indeed a single-source sample, thus providing confidence to single-source calls.

## 4. Conclusions

Increasingly, forensic DNA laboratories are faced with individual evidence items that contain low-level DNA traces from multiple contributors. Capillary electrophoresis of STRs often falls short in the detection and deconvolution of these complex mixture samples, as its ability to detect minor contributors in a mixture relies on the ratio of DNA present from each contributor, the combination of genotypes present, and the total amount of DNA amplified [7]. Further, this information about the contributor status of the DNA sample is only revealed at the end-point of analysis in the traditional workflow, and too often, at that point, reanalysis may be impossible (due to sample consumption) or is extraordinarily labor-intensive (causing delays in processing). Although probabilistic genotyping is a tool increasingly used for mixture identification and deconvolution, it does not provide real-time information before the end of the workflow and the issues previously mentioned will remain if the developed profile does not adequately represent the minor contributor(s). Thus, the ability to discern mixtures versus single-source DNA samples prior to the end-point analysis would be highly valuable to forensic DNA analysts. The developed integrated Quantifiler^TM^ Trio-HRM assay provides a screening tool for the earlier identification of mixture samples at the qPCR step. Furthermore, most modern-day qPCR instruments are constructed with HRM capabilities, thus allowing samples to be fully processed using a single instrument [17,33,34,35,36]. With this coupled capability, the quantification/amplification data can be used to assist in the interpretation of HRM results.

Although the integrated Quantifiler^TM^ Trio-HRM assay exhibited promising results in proof-of-concept testing, studies testing the performance and identifying the limitations of the assay were needed. First, subsequent testing revealed that not one single machine learning tool always outperformed the others for either STR locus tested. Thus, it is recommended that the prediction output from all three prediction models used in this study (SVM radial, SVM linear, and LDA) be considered in the formation of a consensus prediction reported to the analyst. Using this approach would assure that the assay provides the most reliable, and most reproducible, identification of samples as a single source or mixture. In practice, this would look like a prediction output of “single source” or “mix” along with a confidence score between 0 and 1 that indicates the likelihood of that prediction being correct. With the output of a confidence score, labs can establish their own internal confidence cut-off value to determine how to proceed with processing a sample. For example, if a sample is predicted to be a “mixture” with a confidence score of >80%, a lab may proceed with increasing the DNA input into the amplification in order to ensure the DNA from all the minor contributors will be detected, or increasing the capillary electrophoresis injection time for the same reasons [37].

When the best performing model is selected, the integrated Quantifiler^TM^ Trio-HRM assay was reproducible 58.8% of the time across runs and 64.7% within runs. While the samples with DNA concentrations across a broad range (0.01 ng/µL to 60 ng/µL) were predicted accurately for the majority of the samples tested with the integrated Quantifiler^TM^ Trio-HRM assay, the samples predicted to be a single source with concentrations below 0.025 ng/µL or those predicted to be mixtures with concentrations above 0.5 ng/µL were less likely to be accurate and should thus be interpreted with caution. Nevertheless, the optimal performance range of the integrated Quantifiler^TM^ Trio-HRM assay is captured within the linear detection range of the Quantifiler^TM^ Trio kit. The additional studies indicated that the integrated Quantifiler^TM^ Trio-HRM assay was very robust in the identification of the DNA mixture samples, regardless of the number of contributors or the ratio of the major and minor contributors. In fact, the mixtures were more accurately predicted as such as the number of contributors increased. Further, the mixture prediction accuracies were improved well over random chance (6.25%) even when the major/minor ratios were 1:100 or 100:1, which is beyond the sensitivity of traditional CE analysis. Further, the integrated Quantifiler^TM^ Trio-HRM assay allows mixtures with multiple same-sex contributors to be identified at the quantification step, supplementing the information routinely provided by the Quantifiler^TM^ Trio kit. Finally, when the mock evidence samples were evaluated, the prediction accuracies were consistent with previously tested buccal swabs, and all the limitations noted in the reported validation studies held true. It is also important to note that perceptible D5S818 and D18S51 melt curves were produced from these forensic-like samples even when the DNA present was so low that only partial DNA profiles were identified via CE analysis. Taken together, these data display the practical utility and power of the integrated Quantifiler^TM^ Trio-HRM assay. The limitations of this assay that could be addressed in future work include increasing the sample size for each developmental validation study, as well as expanding the selection of developmental validation studies performed. Additionally, further expansion of the training set used for predictions may further improve the prediction capabilities of the assay.

Ultimately, the integrated Quantifiler^TM^ Trio-HRM assay has been proven to be an accurate, inexpensive, and reliable way to gain pertinent information about a sample’s contributor status earlier on in the workflow. As is, this tool can provide forensic examiners with an effective way to screen and triage evidence items prior to the end-point of analysis.

## Figures and Tables

**Table 1:** Confusion matrix output example from prediction modeling algorithm. All samples that had a predicted genotype (within the red box) are considered single-source.

	Predicted Genotype								
**Actual genotype**		10,11	11,11	11,12	11,13	12,12	12,13	13,13	mix
	10,11	5	0	1	1	1	0	0	0
	11,11	1	1	3	1	0	1	0	1
	11,12	1	1	2	3	2	1	1	1
	11,13	0	1	1	3	0	0	0	1
	12,12	0	0	1	1	4	0	0	2
	12,13	1	0	0	0	2	1	1	3
	13,13	0	0	2	1	0	1	1	1
	mix	1	0	2	4	0	1	0	8

**Table 2:** Inter-run reproducibility of single-source and mixture samples assessed with the integrated Quantifiler^TM^ Trio-HRM assay.

	Single-Source Reproducibility (n = 7)	Mixture Reproducibility (n = 10)	Overall Reproducibility (n = 17)
Previously recommended models (SVM radial, [16])	71.4%	30.0%	47.1%
Best performing models (SVM linear and LDA)	85.7%	40.0%	58.8%

**Table 3:** Sensitivity assessment for single-source and mixture samples tested across a range of DNA concentrations *.

	Single Source	Mixture
Sample Concentration (ng/µL)	Number Correctly Predicted	Number Correctly Predicted
60	5/5	2/5
50	5/5	1/5
5	4/5	1/5
0.5	5/5	3/5
0.05	3/5	4/5
0.025	3/5	4/5
0.01	0/5	5/5

* Red line indicates optimal range.

**Table 4:** Prediction accuracies for two-person (2p), three-person (3p), four-person (4p), five-person (5p) mixtures.

Number of Contributors	Number Correct	Prediction Accuracy
2p	13/16	81.0%
3p	4/5	80.0%
4p	5/5	100.0%
5p	5/5	100.0%
	Overall prediction accuracy	87.1%

**Table 5:** Prediction accuracies for mixture ratios ranging from 1:1 to 1:100.

Mixture Composition	Number Correct	Prediction Accuracy
1:1	15/16	93.8%
1:2/2:1	15/20	75.0%
1:5/5:1	17/20	85.0%
1:10/10:1	18/20	90.0%
1:20/20:1	11/16	68.8%
1:50/50:1	9/16	56.3%
1:100/100:1	8/16	50.0%
	Overall prediction accuracy	75.0%

**Table 6:** Average small autosomal quantification value and degradation index of mock evidentiary samples tested in the standard Quantifiler^TM^ Trio assay and Integrated Quantifiler^TM^ Trio-HRM assay.

	Quantification Value ^ (n = 17)	Degradation Index * (n = 17)
Standard Quantifiler^TM^ Trio assay	0.1741	1.64
Integrated Quantifiler^TM^ Trio-HRM assay	0.2121	1.17

^ *p* = 0.05, * *p* = 0.01.

**Table 7:** Predictions of mock case-type samples that were collected from different sources aged over a varying number of simulated days [26].

Sample	DNA Source	Simulated Days	Concentration (ng/µL)	Known Status	Number of Contributors	Correct Prediction
BB14C1	Blood	1	0.1263	Single-source	1	yes
32-313	Blood	413	0.3754	Single-source	1	yes
32-315	Blood	413	1.2087	Single-source	1	no
32-319	Blood	413	0.3959	Single-source	1	no
16-157	Blood	1440	0.2836	Single-source	1	no
16-159	Blood	1440	0.2409	Single-source	1	yes
TB2H1	Wearer	68	0.0236	Single-source	1	yes
18-172	Wearer	180	0.1053	Mixture	2	yes
30-291	Wearer	413	0.1083	Mixture	3	*
30-292	Wearer	413	0.1414	Mixture	2	yes
30-296	Wearer	413	0.0603	Mixture	3	yes
17-161	Touch	180	-	Single-source	1	no
KB1F	Touch	240	-	Single-source	1	no
KB3F	Touch	240	0.0541	Mixture	3	yes
KB4F	Touch	240	0.1920	Mixture	2	yes
31-301	Touch	413	0.1217	Single-source	1	no
31-302	Touch	413	0.0361	Mixture	3	yes
28-276	Touch	1440	-	Mixture	3	yes
28-277	Touch	1440	0.0542	Single-source	1	no
28-278	Touch	1440	0.0786	Mixture	2	yes
		Overall prediction accuracy				63.2%

- represents samples not used in quantification assessment. * represents samples not used in prediction assessment.

## Data Availability

The datasets generated during the current study are available from the corresponding author upon reasonable request.
